# Enhancing the Flexibility of TCP in Heterogeneous Network

**DOI:** 10.1371/journal.pone.0161249

**Published:** 2016-09-22

**Authors:** Wang Hui, Li Peiyu, Fan Zhihui, Li Zheqing, Wei Xuhui

**Affiliations:** 1 Network Information Center of Henan University of Science and Technology, Luo Yang, 471003, China; 2 Information Engineering College of Henan University of Science and Technology, Luo Yang, 471003, China; Beihang University, CHINA

## Abstract

Due to a set of constant initial values, the performance of conventional TCP drops significantly encountering heterogeneous network, showing low throughput and unfairness. This paper firstly demonstrates the chaotic character of TCP congestion control in heterogeneous network, especially the sensitivity to initial value. Inspired by merit of nature-inspired algorithm, a novel structure of TCP congestion control (IPPM, Internet Prey-Predator Model) is proposed. Parameters such as available link capacity(*C*), congestion window (*W*) and queue length (*Q*) are collected by IPPM, which calculates the max value of *C* according to the interacting relationship existing in *C*, *W* and *Q*, and IPPM initiates the TCP ssthresh with the calculated value. Plenty of simulation results show that the modified TCP can effectively avoid network congestion and packet loss. Besides, it holds high resource utilization, convergence speeds, fairness and stability.

## 1 Introduction

Since existing high wireless error, long transmission delay and complex network structure in heterogeneous network, the performance of traditional TCP is limited seriously as TCP is designed for wired network [1–2]. We analyze the reason of problems mentioned above before and after packet loss. Before the packet loss, the traditional TCP presents low adaptability and poor flexibility. As we all know, TCP has many constant initial values when the transmission starts. For example, the initial value of cwnd is set as 1 mss(maximum segment size) and the initial value of ssthresh is set as 65535 bytes. Because of the stable environment in wired network, constant values didn’t limit the performance of TCP too much, but in heterogeneous network, this will cause many problems. For instance, the value of ssthresh, which is the threshold between SS(slow start) phase and CA(congestion avoidance) phase. On one hand, if the initial value of ssthresh is relatively small in the environment of large bandwidth, small delay and light link load, then TCP flow will end the SS phase and step into the CA phase prematurely which will cause low link utilization. On the other hand, if the ssthresh is too large for current link state, the sender will enhance the sending rate which may cause link congestion and packet loss. After the packet loss, traditional TCP can’t distinguish the specific reason of packet loss. In wired network, most packet losses are caused by congestion, but in wireless or heterogeneous networks, BER is another important reason of packet loss which can also cause performance degradation of TCP [3].

To solve the problem of poor flexibility caused by constant initial settings, we assume that a dynamic and appropriate setting of initial value will make the complex network transmission change to an easy-handled system. As a precondition, TCP transmission must be sensitive to the initial value. It is generally known that sensitive to the initial value is an important characteristic of chaos system. So we should confirm whether TCP protocol behaves chaotically in heterogeneous network. In recent years, a large body of researches on analyzing the nature of TCP protocol has been published in literatures. Zhao used phase-space reconstruction in chaos theory to detect covert channel in TCP initial sequence numbers and solved the problem of testing covert channel [4]. JS. Wang verified the bifurcation and chaos character in RED-AQM, and proved the existence of communication delay threshold to determine whether the system is stable [5]. Veres pointed out that the congestion control of TCP performs certain periodicity and predictability, and confirmed the chaotic nature of TCP in wired network [6]. To our best knowledge, the chaotic nature of TCP congestion control in heterogeneous network hasn’t been confirmed yet and there are no studies involved in using chaotic theory to improve the performance of TCP in heterogeneous network. Next section will demonstrate the chaotic nature of TCP congestion control in heterogeneous network.

In order to calculate the initial value more scientifically and intelligently and improve the flexibility of TCP in heterogeneous network, we employ the idea from nature-inspired algorithm. Nature-inspired algorithm has characters such as self-organization, robustness, scalability and adaptability, which are highly desired by complex network environment [7]. Besides, nature-inspired algorithms have been applied in many areas such as network security, pervasive computing, sensor network and artificial intelligence which had made remarkable achievements [8–9]. After the analysis of nature-inspired algorithms and congestion control mechanisms, we propose a novel structure of TCP congestion control model based on prey-predator algorithm [10]. In this model, TCP parameters are initialized with calculated values, which will make the TCP stay in an effective operating state and utilize the available network bandwidth maximally.

The rest of this paper is organized as follows. In section 2 the chaotic characteristic of TCP congestion control in heterogeneous network is analysed and verified. In section 3 the theoretical basises and structure of IPPM are presented. Section 4 is dedicated to analyzing the parameters of proposed model. In section 5 the simulation results are exposed and discussed, focusing on the throughput, fairness and packet loss rate of IPPM. At last, we make a summary of this paper as well as the future work.

## 2 Discussion

**Fig 1:**
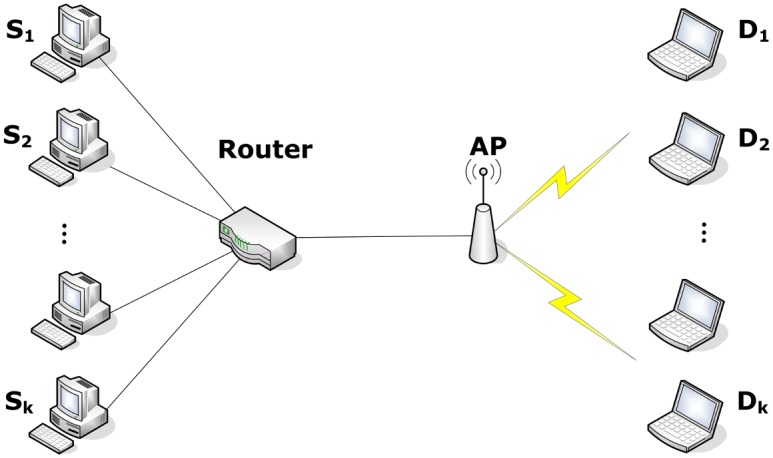
The topological graph of Heterogeneous Network.

If a system shows the characteristics of periodicity, strange attractor and initial value sensitivity, we confirm this system as a chaotic system [11]. Next, we will find out whether does TCP congestion control in heterogeneous network hold these characteristics using NS2. The network environment is shown as Fig 1, where, *S* denotes sender, *AP*(Access Point) represents the last jump of the wireless access point, *D* indicates the wireless receiver.

### 2.1 Strange Attractor of Chaotic Nature

Strange attractor is a production in chaotic system which reflecting the motional characteristic of disorderly steady state, and it’s also a kind of motion morphology of chaotic system. We can prove the chaotic nature of the system through analyzing the attractor of one dimension [12].

Because cwnd is just one of the numerous parameters in the whole system, and not continuous, which means that cwnd cannot describe the properties of a complex system effectively, especially the hidden nature of the system. It is particularly important to expresses the current operating condition of the system more accurately. Literature [13] proposed a time shift algorithm to reconstruct the phase space of the parameter, so as to show some hidden properties in complex system effectively. The algorithm is shown as follows:

$$x[i]=\frac{1}{n}\sum_{j=1}^{n}\text{cwn}{\text{d}}_{x}[i-j]$$ (1)

$$y[i]=\frac{1}{n}\overset{n}{\underset{j=1}{\sum }}\text{cwn}{\text{d}}_{y}[i-j]$$ (2)

where *x* and *y* represent two TCP flows. *n* denotes the range of control statistics, a bigger value of *n* indicates a better reconstruction. This method holds two advantages:

1) Let *w* represent the number of cwnd, the possible points of cwnd phase space will increase from *w*^*2*^ to *(nw-n)*^*2*^ using this method.

2) The distance between *x[i]* and *x[i+1]* becomes smaller using this method, which should exactly not more than (2*w-2)/n. So this time shift algorithm can express the property of system more accurately and continuously.

**Fig 2:**
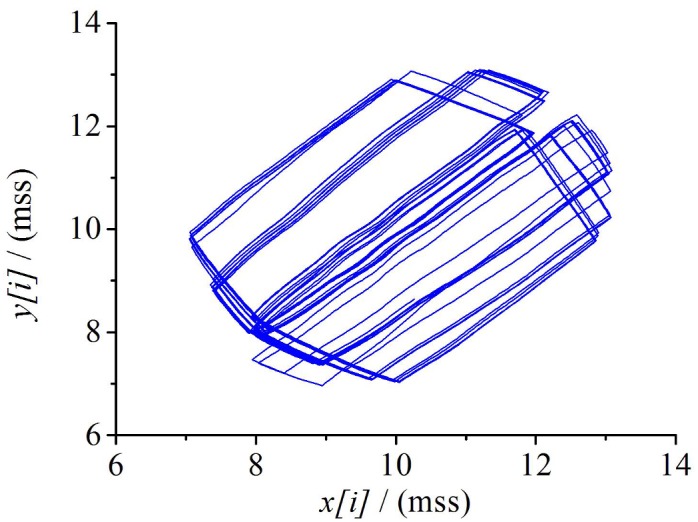
The attractor in cwnd phase space.

We set the initial conditions of system as follow: the bandwidth of bottleneck link (*BB*) = 0.5Mbps, the link delay(*LD*) = 10ms, and queue length(*QL*) = 20packets, range of control statistics (*n*) = 100. Data sends from *S*_*1*_ and *S*_*2*_ through wired link, and then they will be send from *AP* to *D*_*1*_ and *D*_*2*_ respectively. Two TCP flows are expressed as *TCP*_*0*_ and *TCP*_*1*_, the size of sending window is respectively labelled as *cwnd(0)* and *cwnd(1)*. After using the above algorithm to reconstruct the phase space of cwnd, we can get Fig 2, where, x*[i]* and *y[i]* represent a pair of values produced by time shift algorithm at a certain moment. Fig 2 indicates that the attractor appears after a sufficient running time, and it performs steady in some plausible disordered status.

The common method to confirm a attractor as a strange attractor is to find out whether the attractor has fractal structure [14]. The fractal dimension of the attractor can be calculated by the method of box dimension as following. Assuming that *S* represents the square length of Δ, *F* is a non-empty and bounded set, and *N (F)* denotes the number of square where *F* intersects with *S*. Then the box dimension of above attractor *D (F)* can be expressed as follows:

$$D(F)=\underset{\Delta \to 0}{lim}\frac{lgN(F)}{lg(1/\Delta )}$$ (3)

y=kx+b (4)

In Eqs(3) and (4), we consider that the linear relation between *y* and *x* are *y = lgN(F)* and *x = lg(1/Δ)*. *k* is the slope of *lgN(F)~lg(1/Δ)* and b is intercept. We can get the box dimension of this attractor *D(F) = k≈*2.3371 after fitted by the least-square method. This box dimension is non-integer dimension, indicating that the attractor has a fractal structure which we called strange attractor. The strange attractor is one of the main characteristics of chaos, so it contradicts our hypothesis, and therefore proves the result.

### 2.2 Sensitivity to Initial Value of Chaotic Nature

**Fig 3:**
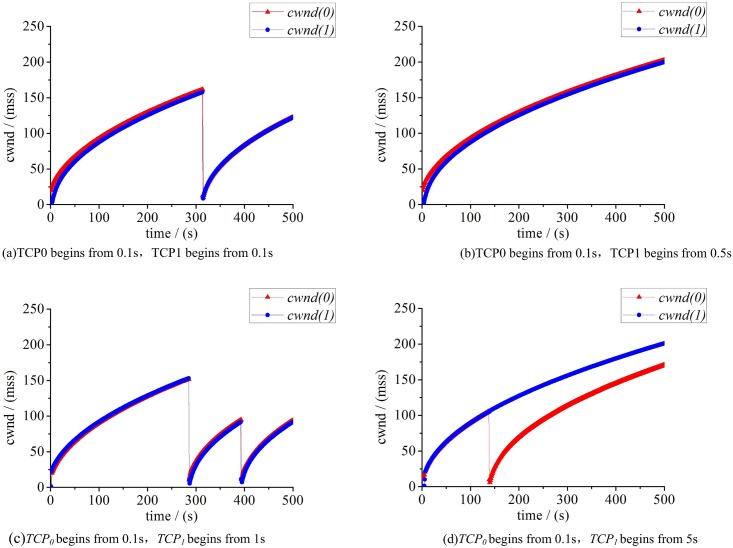
Two TCP flows with different start time.

The initial value sensitivity refers to a minor change at the beginning which results in a tremendous difference in the end. It is one of the important properties of chaotic system, namely, butterfly effect. Next, we will analyze the initial value sensitivity of TCP congestion control in heterogeneous, considering the bandwidth of the bottleneck link(*BB*) = 2Mbps and the other conditions remain unchanged. Simulation results are compared among four groups of experiments. In each group, *TCP*_*0*_ begins to send data from 0.1s, while *TCP*_*1*_ starts from 0.1s, 0.5s, 1s and 5s respectively, both of them records the value of cwnd per 10ms. Experimental results are shown in Fig 3.

Fig 3 shows the changes of cwnd in the scenarios of two flows when starting at different time. Obviously, the subsequent evolutions of system show extreme different forms after a tiny change of initial condition. In a nutshell, the cwnds of two flows present irregularity and initial value sensitivity which meet the property of chaos system. What’s more, in the case of Fig 3(b), we can find that the system works well with no packet loss, and two flows keep a high sending rate synchronously. While in other cases, the system has a different degree of congestion or loss. Intuitively, the system in Fig 3(b) becomes more simple and efficient which can prove that a proper initial value can make a complex system change to a relatively simple and efficient system.

Further proving the initial value sensitivity of TCP in heterogeneous network, we increase the number of TCP flows to 30, specifically, *BB* = 1Mbps, *LD* = 15ms, *QL* = 60 packets. When the running time arrived at 50s, one of the flows is choosed randomly and it’s cwnd is increased by 1, then we compared the system with unchanged one. To show the differences between these two systems intuitively, we define the Euclidean distance of cwnd in phase space as follow:

$$Ed(t)=\sqrt{\sum_{i=1}^{N}{\left(w^{orig}(i,t)-w^{pert}(i,t)\right)}^{2}}$$ (5)

**Fig 4:**
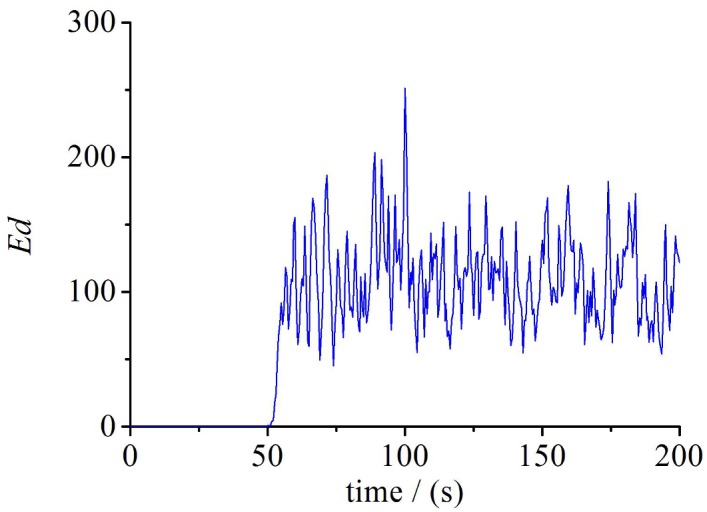
The difference between the original and modify system.

In formula (5), *w*^*orig*^(*i*,*t*) represents the cwnd of the flow*(i)* at time t in the original system, *w*^*pert*^(*i*,*t*) represents the value of cwnd of the flow*(i)* at time t in the changed system. N denotes the number of TCP flows, here, we set N = 30. Fig 4 shows the difference between two systems in phase space of cwnd over time. The Euclidean distance of two systems in the cwnd’s phase space is 0 before changed (before 50s), indicating that the two system is exactly the same. Due to the slightly change made at the 50s(increasing the cwnd of one flow), the subsequent evolution of the system is completely different compared with before. It suggests that the system has the butterfly effect, and the butterfly effect is one of important index of the chaotic system. Given these features, it should come as no doubt that TCP congestion control has chaotic nature in heterogeneous network.

## 3 Models

According to the previous section which verified the chaotic characteristic of TCP congestion control in the heterogeneous network, we could make a conclusion that a set of appropriate parameters can lead the heterogeneous system converge to a stable and balanced state with high bandwidth utilization. To achieve this goal, an internet prey-predator model inspired by nature is proposed. The reason we choose predator-prey model algorithm is that the predator-prey model has a high logical mapping relationship with TCP congestion control mechanism(introduced in next section). Moreover, nature-inspired algorithm has a strong advantage in solving the problem in complex system.

### 3.1 Prey-Predator Model

In the food chain of ecosystem, we assume that *a* and *b* represent prey and predator respectively, *N(a)* and *N(b)* denote the number of each species. The interaction relationships between *a* and *b* can be explained by Eqs (6) and (7)[6]:

$$\frac{dN(a)}{dt}=\alpha (1-\frac{N(a)}{C_{N(a)}})N(a)-\beta N(a)N(b)$$ (6)

$$\frac{dN(b)}{dt}=\chi N(a)N(b)-\delta N(b)$$ (7)

where *α* is the natural growth rate of *a*,*β* is the death rate when *a is* preyed upon by *b*. *χ* represents the increasing rate of *b* after praying on *a*, *δ* is the nature death rate of *b*. *C*_*N(a)*_ refers to the carrying capacity of *a* in the absence of *b*, which means the max value of *N(a)*. This is a two level prey-predator model, and it can be extented to a three level model as shown in Eqs (8)–(10). Where *ε* is the death rate of b when it is preyed upon by c. *φ* is the increasing rate of *c* after praying on *b*. *γ* represents the nature death of *c*.

$$\frac{dN(a)}{dt}=(\alpha (1-\frac{N(a)}{C_{N(a)}})-\beta N(b))N(a)$$ (8)

$$\frac{dN(b)}{dt}=(\chi (1-\frac{N(b)}{C_{N(b)}})+\delta N(a)-\varepsilon N(c))N(b)$$ (9)

$$\frac{dN(c)}{dt}=(\varphi N(b)-\gamma )N(c)$$ (10)

In the three level prey-predator model, when the number of *b* reduces sharply, *a* will grow exponentially, so *b* has played an important role to reduce the growth of *a*. On the other hand, when the number of *a* reduces rapidly and substantially, *b* will decline exponentially, the interaction relationship between *a* and *b* shows character of ratio dependencies. Besides, *a* has a highest carrying capacity in the absence of *b*. Similarly, the relationship between *c* and *b* is the same to *a* and *b*.

### 3.2 Internet Prey-Predator Model

**Fig 5:**
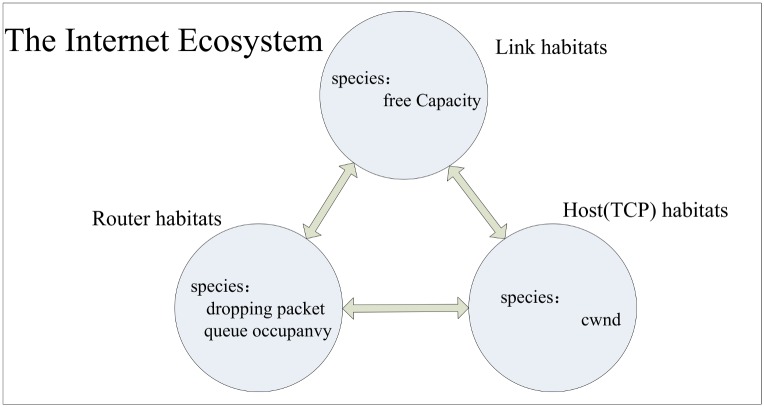
Internet ecosystem.

We consider the heterogeneous network as an ecological system where lives species like cwnd, queue length, and link capacity. The values of parameters represent the number of each species which connected and influenced with each other, and the relationships between cwnd, available link capacity and queue length can be described as follows:

(1) First, for available link capacity(*C*) and cwnd(*W*), when packet sending rate declines, available link capacity will grow and vice versa. On the other hand, if there is any growth of available link capacity, the sending rate will rise and vice versa. Moreover, the bandwidth in the bottleneck link also limits the max value of the available link capacity.

(2) Second, for queue length(*Q*) and *W*, when lacking of waiting packet, cwnd will grow and vice versa. When packet sending rate declines, the queue length will decline and vice versa. The capacity of link limits the max value of packet sending rate.

(3) It is obviously that the mutual restriction relations between *C*, *W* and *Q* conform to the logic of prey-predator model. In other word, we can use prey-predator model to locate congestion problem in heterogeneous network, and the internet ecosystem is described as Fig 5.

According to the three level prey-predator model, internet prey-predator model is explained as Eqs (11)–(13),

$$\frac{dC}{dt}=C(\alpha (1-\frac{C}{C_{c}})-\beta \sum_{j=1}^{k}W_{j})\sigma$$ (11)

$$\frac{dW_{i}}{dt}=W_{i}(\chi_{i}(1-\frac{W_{i}}{C_{W}})+\delta C-\varepsilon Q)\;i=1,\text{…‥}k$$ (12)

$$\frac{dQ}{dt}=Q(\sum_{i=1}^{k}\varphi_{i}w_{i}-\gamma )\sigma \;\gamma =M\text{in}(BB,Q+\sum W_{i})$$ (13)

where *σ* is a positive smooth factor and *W*_*i*_ represents the value of cwnd, *C*_*c*_ and *C*_*w*_ are the max value(carrying capacity) of *C* and *W*, *α*, *β*, *χ*, *δ*, *ε*, *φ*, *γ* have been introduced above. *Q* can be calculated as follow equation, where the Packetsize is 1040Byte.

$$Q=\frac{BB*RTT*1000}{Packetsize*8}$$ (14)

### 3.3 Congestion Control Algorithm

In this paper, we divide TCP congestion control into two phases: before packet loss and after packet loss. Preventing network congestion is the main task of IPPM before packet loss which we will illustrate below. After the loss we can distinguish the reason of packet loss and take the corresponding measures to deal with congestion loss and wireless loss, which is not the main content of this paper.

**Fig 6:**
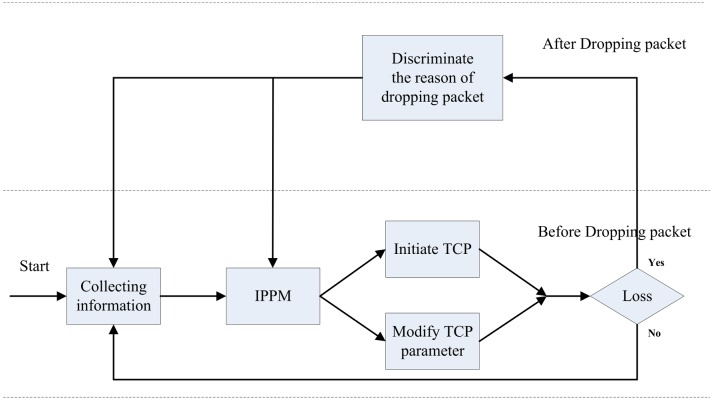
The structure of improved TCP congestion control mechanism.

When TCP flow starts, the sender collect parameters from the network, and compute a proper value of cwnd using IPPM to initiate parameter dynamically. In this way, TCP flow can run with an optimum state and enhance the adaptability encountering different environments and transmission state, so as to avoid congestion occurrence as well as dropping packets. The processes of collecting and adjusting are conducted periodically, which can improve the adaptability of TCP and take corresponding adjustment according to the dynamic change of network. The structure of proposed TCP congestion control algorithm is depicted as Fig 6.

## 4 Results

**Fig 7:**
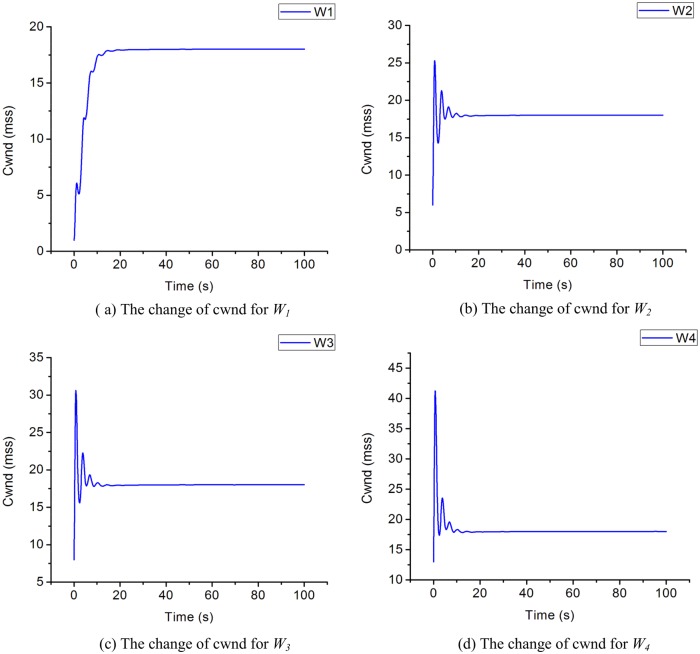
The evolution for cwnd in proposed algorithm. (a) The change of cwnd for W1. (b) The change of cwnd for W2. (c) The change of cwnd for W3. (d) The change of cwnd for W4.

**Fig 8:**
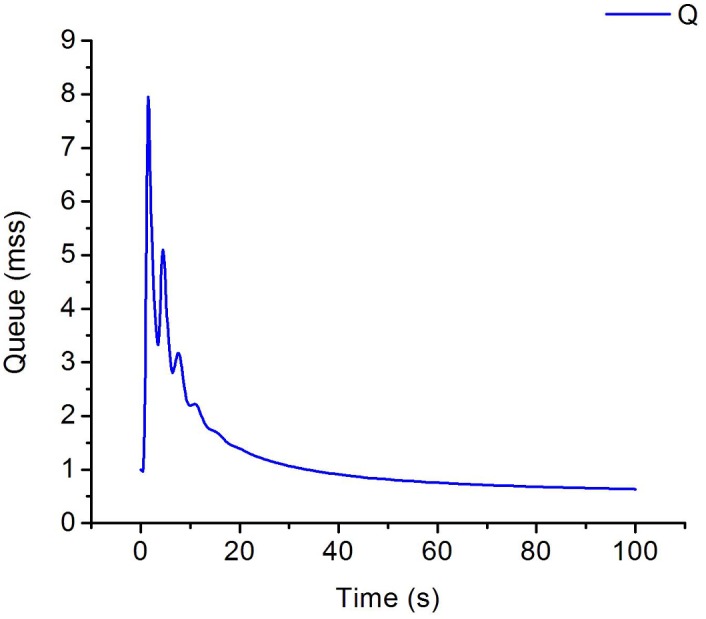
The evolution of queue for bottleneck link.

**Fig 9:**
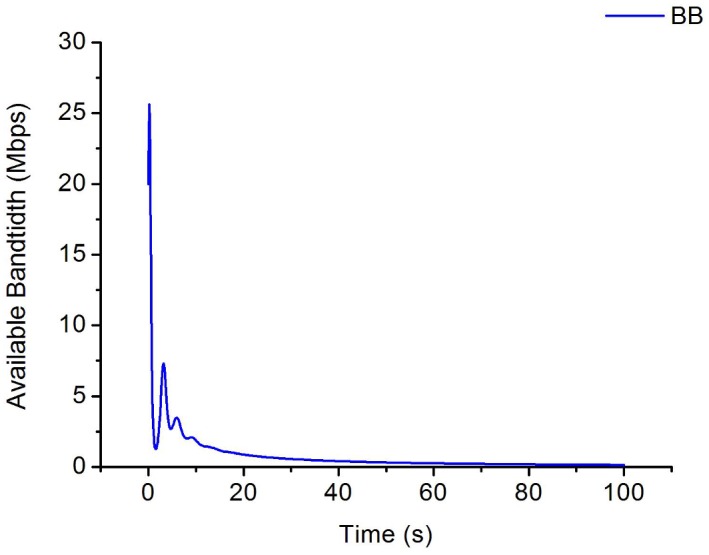
The evolution of available bandwidth for bottleneck link.

In this section, we analyze and verify the changing trends of C, W and Q, then, we evaluate the performance of IPPM. Consider scenarios with four flows send from *S*_*1*_*~S*_*4*_ respectively, and the initial values of cwnds are set as: *W*_*1*_ = 1, *W*_*2*_ = 6, *W*_*3*_ = 8, *W*_*4*_ = 13, specifically, other parameters are set as: *α = C*_*c*_ = *C*_*w*_ = *BB*, *β* = 1, *ε* = *δ* = *σ* = 0.1, *χ*_*i*_ = 0.5, *BB* = 20Mbps, *LD* = 30ms, packet size = 1040B. Simulations run under the topology Fig 1 and Eqs (11)–(14) are used to plot the change of *Q*, *BB* and *W*_*1*_*~ W*_*4*_. The results are shown as Figs 7–9.

From Fig 7, we can draw a conclusion that even though *W*_*1*_*~W*_*4*_ are set with different initial value, they can finally convergence to a balanced state after running a period of time, moreover, the sending rate of all flows are almost the same. So IPPM is a cure for the traditional TCP with defects of unfairness.

As is evident from Fig 8, the queue length of bottleneck link increases sharply at the first 5s then achieves a steady-state to almost 0 at last which means there is no packets stacking in the bottleneck link and all packets are transmitted fluently. Fig 9 shows the change of available bandwidth of bottleneck link, we can find that available bandwidth is almost near to 0 at last, which means the utilization of bandwidth is almost up to 100%.

Next, we evaluate the performance of throughput, fairness and packet loss rate of IPPM. As we all know, ssthresh is the threshold of cwnd between SS and CA phase, where the cwnd increase exponentially before while linearly after. So how to choose an appropriate value for ssthresh is crucial important. This is the reason why we choose ssthresh as the parameter to be initialized so as to improve the performance of conventional TCP. Experiments are finished in NS2 using topology shown in Fig 1, and results are compared with the traditional TCP. We consider scenarios with *BB* = (10, 20Mbps), *LD* = (10, 20, 30 ms), and the number of flows = (2,4,8). The values of ssthresh computed by IPPM in different conditions are shown as Table 1.

**Table 1:** ssthresh value computed by IPPM.

Bandwidth (mb)	Delay (ms)	Flow number	ssthreshValue	Bandwidth (mb)	Delay (ms)	Flow number	ssthreshValue
10	10	2	6.00	20	10	2	12.00
		4	3.01			4	6.01
		8	1.50			8	3.00
	20	2	11.5		20	2	23.00
		4	6.01			4	12.03
		8	3.00			8	6.00
	30	2	16.38		30	2	34.32
		4	9.01			4	18.02
		8	4.50			8	9.00

**Fig 10:**
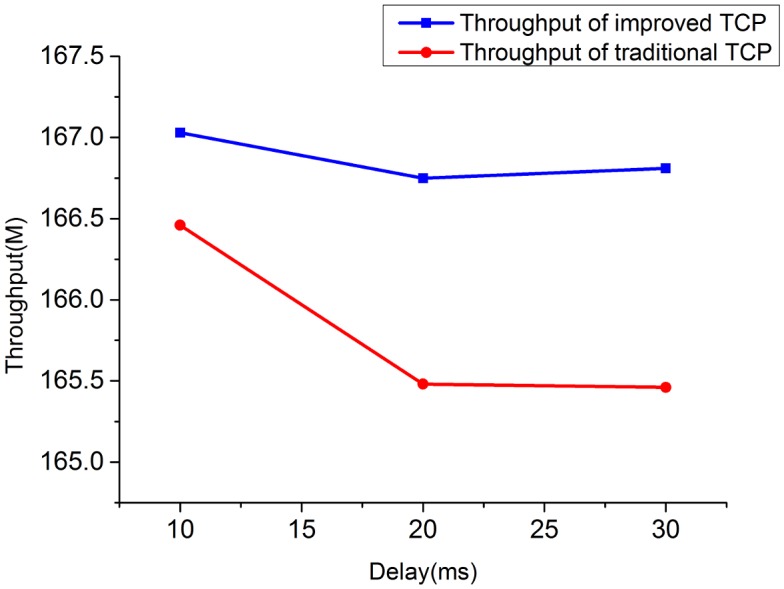
The comparison of throughput for 2 TCP flows when bandwidth = 10M.

**Fig 11:**
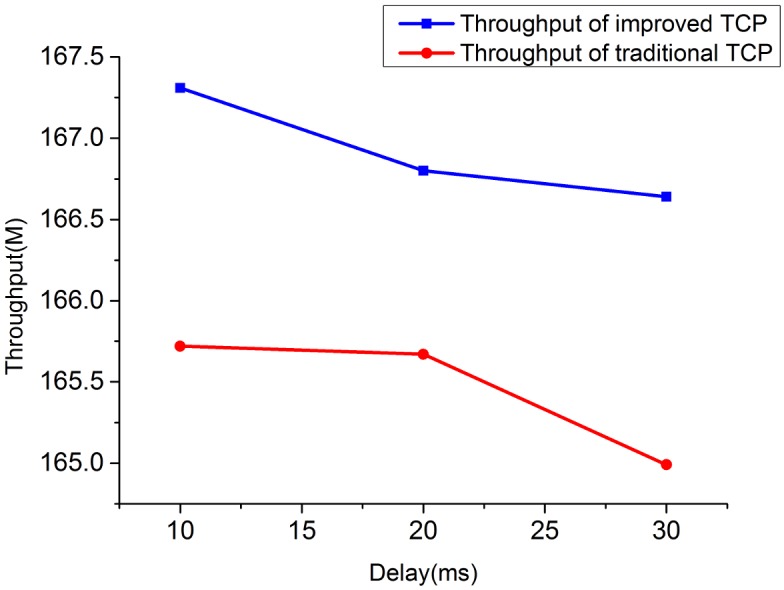
The comparison of throughput for 2 TCP flows when bandwidth = 20M.

**Fig 12:**
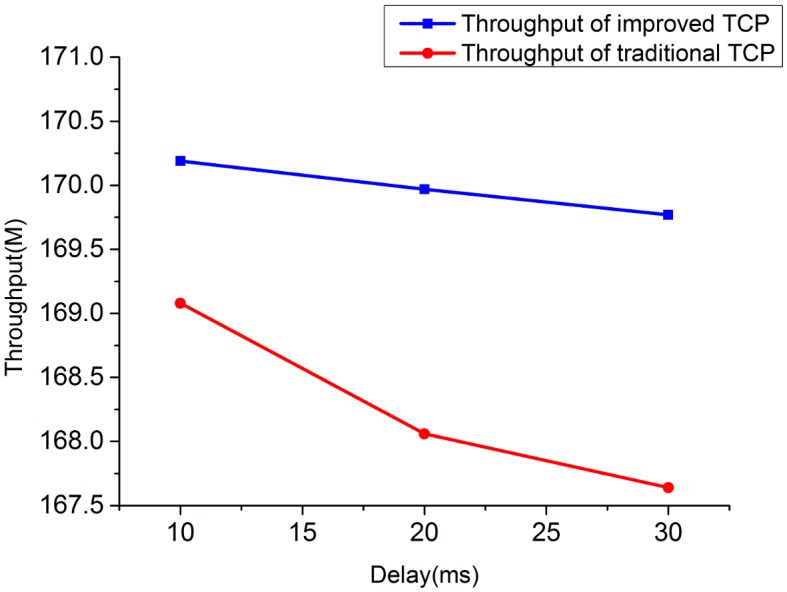
The comparison of throughput for 4 TCP flows when bandwidth = 10M.

**Fig 13:**
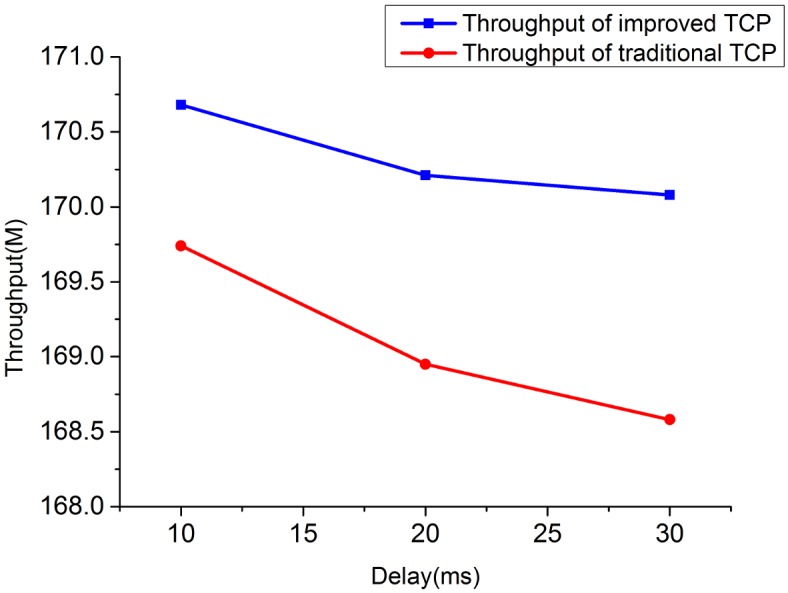
The comparison of throughput for 4 TCP flows when bandwidth = 20M.

**Fig 14:**
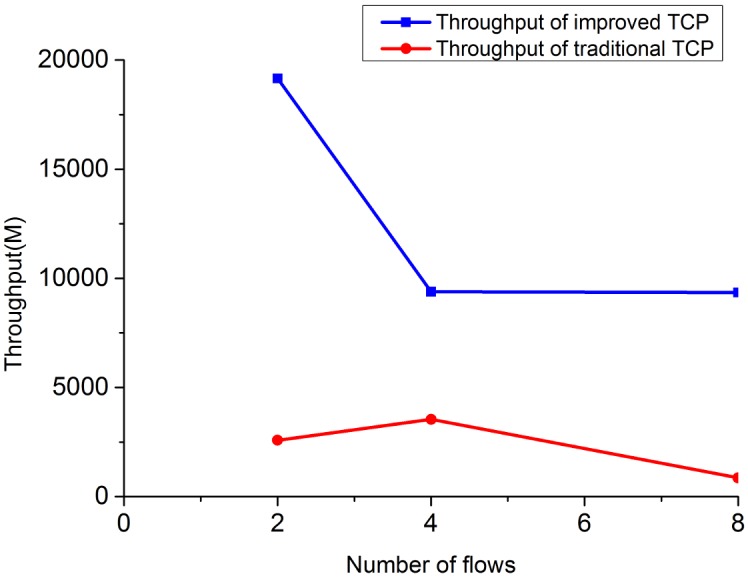
The comparison of throughput for high bandwidth-delay product network.

**Fig 15:**
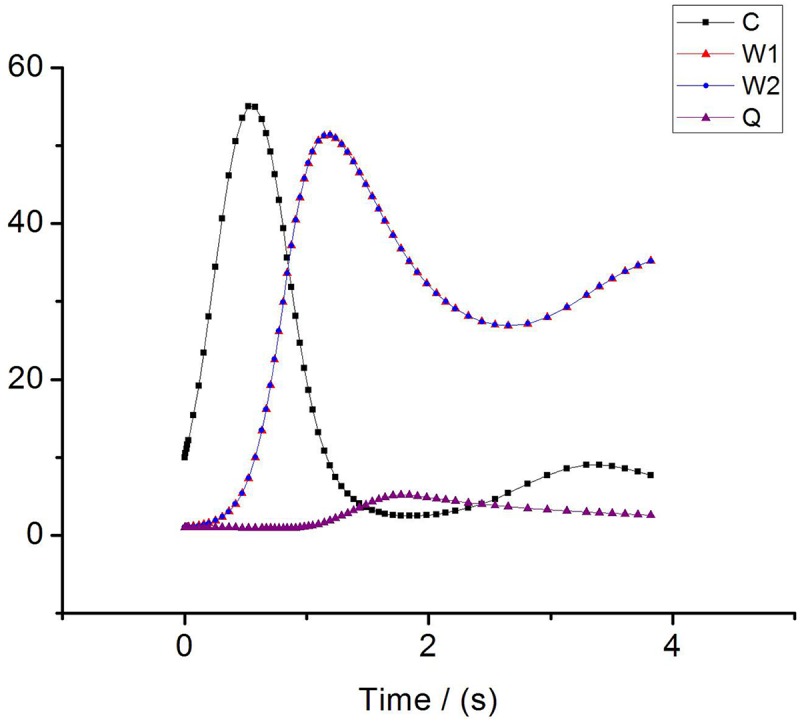
The fairness of two flows.

**Fig 16:**
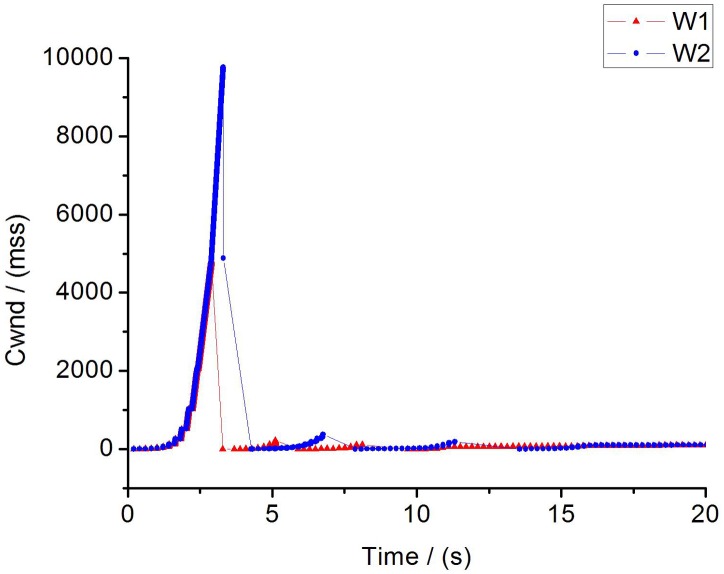
The changing of cwnd for traditional TCP.

**Fig 17:**
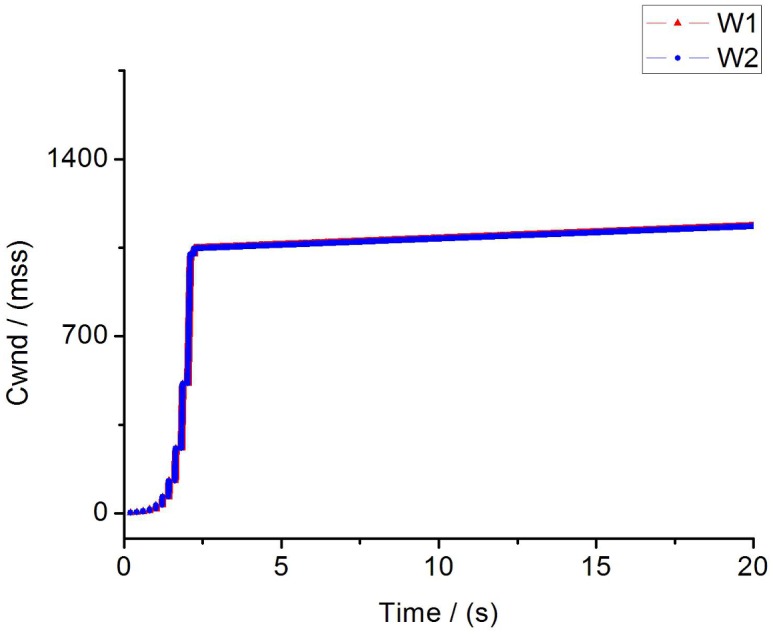
The changing of cwnd for improved TCP.

To give readers an intuitive understanding of the performance improvement of IPPM, we compare the throughput of improved TCP with traditional TCP under heterogeneous network and high bandwidth-delay product network, we also evaluate the fairness and packet loss properties between proposed TCP and conventional TCP, and results are showing as Figs 10–17:

1) Throughput under heterogeneous network: Figs 10–13 show the throughput comparison between improved TCP and traditional TCP under scenarios of 2 and 4 flows each. As is evident from these figures, throughput of improved TCP is larger than the traditional TCP, and with the increment of bandwidth-delay product, the improvement of throughput is more outstanding.

2) Throughput under high bandwidth-delay product network: to eliminate the limitation of wireless and create a network with large bandwidth and long delay, we choose the wired topology to simulate the experiment. The configurations simulated here are set as: *BB* = 100Mbps, *LD* = 100ms. Fig 14 shows the throughput comparison between improved TCP and traditional TCP under scenarios of 2, 4 and 8 flows in large bandwidth-delay product environment. The throughput of improved TCP is 19156.68M, which is almost 6 times of the traditional TCP’s 2582.36M.

3) Fairness performance: we introduce an equation named fairness index as shown in Eq 15. Where *m* denots the number of users involved in computing the fairness index, and *x*_*i*_ represents the resource owned by user *i*. The value of fairness index is ranging from 0 to 1, the closer the index to 1, the better fairness it is [15].

$$F\left(\text{x}\right)=\frac{{\left(\sum x_{i}\right)}^{2}}{m(\sum x_{i}^{2})}$$ (15)

We calculate the fairness index of each flow using the above equation in the scenario of two flows. The fairness index of traditional TCP flow is 0.836. In contrast, the fairness index of improved TCP flow is 0.997 which is almost close to 1. Obviously, the fairness of improved TCP is much better than conventional TCP. Furthermore, to evaluate the TCP fairness through different parameters, we consider a 100M bottleneck link with round-trip delay(RTT) of 100ms. Simulation results are shown as Table 2 and Fig 15. In Table 2 we find that the throughput of flow1 and flow2 are almost the same in case of improved TCP, which means these two flows share link resources equally. While, in traditional TCP they are dramatically different. In Fig 15 it can be easily observed that four parameters converge to a steady state gradually under the constraint of IPPM. More importantly, *W*_*1*_ is overlap with *W*_*2*_, so there is no strong TCP or weak TCP, and two flows are allocated with fairly sending rate and bandwidth utilization.

**Table 2:** The comparison of throughput for 2 flows.

	Throughput of Flow1/Mb	Throughput of Flow2/Mb
**Improved TCP**	9578.35	9578.33
**Traditional TCP**	999.90	1582.46

4) Packet loss performance: Fig 16 shows the changing of cwnd under the traditional TCP, we can find that W_1_ encounters packet loss at 2.8s and W_2_ loss packet at 3.2s. After packets loss, senders cut down their cwnd to 1, then stay in CA phase until the end of simulation. Unfortunately, the sending rates of two flows maintain at a low level as well as the bandwidth utilization during the stage of CA. The statistics in Table 3 show that the number of packet loss is up to 4835 in traditional TCP which is the reason why traditional TCP degenerate seriously.

**Table 3:** The comparison of dropping packet for 4, 8 flows.

Number of flow	The times of dropping packet for traditional TCP	The times of dropping packet for improved TCP
4	3104	0
8	4786	8

While in our efforts as is shown in Fig 17, the cwnds of these two flows increase to 1000 exponentially at first 4s and step into CA phase until the end. There is no packet loss and the sending rate remains at a high level during simulation.

As we all know, when the packet loss occurred in the traditional TCP, the sender will slow down its sending rate sharply, especially in the fully congestion situation, in order to alleviate the congestion, the cwnd will be set as 1 immediately. In our simulations of traditional TCP, when the first packet loss happens, the high sending rate will lead to consecutive packets losses. As a result, senders will re-transmit the dropping packet and remain at a small sending rate in the CA phase, and that’s the reason why traditional TCP performance degraded significantly. Since IPPM could prevent the packet loss, it could improve the performance of conventional TCP. Given these features, it should be no doubt that a significant contribution to improve the performance of conventional TCP in reducing the packet loss has been made by IPPM.

## 5 Conclusion

In this paper, we address the problem of TCP performance degeneration in heterogeneous network with IPPM congestion control algorithm. In particular, we find the chaotic nature of TCP in heterogeneous network, especially the sensitivity to initial parameter. Then, we propose a novel structure of TCP congestion control combined with the merit of nature-inspired algorithm, which can initiate the TCP flexibly using internet pray-predator model and lead the network converge to a stable and balanced state with high bandwidth utilization quickly. Numerical results in different situation show that the proposed algorithm manifests high bandwidth utilization, better astringency and fairness.

An interesting direction for further work is developing the IPPM with more related parameter such as loss rate, bit error rate and RTT to enrich the model. Moreover, we plan to transplant the IPPM algorithm to the real network environment and make deep research in improving the performance of TCP with chaotic control theory.

## Supporting Information

S1 File Replication of Fig 1. (ZIP)

S2 File Data for Fig 2 and replication of Fig 2. (ZIP)

S3 File Data for Fig 3 and replication of Fig 3. (ZIP)

S4 File Data for Fig 4 and replication of Fig 4. (ZIP)

S5 File Replication of Fig 5. (ZIP)

S6 File Replication of Fig 6. (ZIP)

S7 File Data for Fig 7 and replication of Fig 7. (ZIP)

S8 File Data for Fig 8 and replication of Fig 8. (ZIP)

S9 File Data for Fig 9 and replication of Fig 9. (ZIP)

S10 File Data for Fig 10 and replication of Fig 10. (ZIP)

S11 File Data for Fig 11 and replication of Fig 11. (ZIP)

S12 File Data for Fig 12 and replication of Fig 12. (ZIP)

S13 File Data for Fig 13 and replication of Fig 13. (ZIP)

S14 File Data for Fig 14 and replication of Fig 14. (ZIP)

S15 File Data for Fig 15 and replication of Fig 15. (ZIP)

S16 File Data for Fig 16 and replication of Fig 16. (ZIP)

S17 File Data for Fig 17 and replication of Fig 17. (ZIP)
