# Supplementary figures and images for: Enhancing the Flexibility of TCP in Heterogeneous Network
Source: PLoS One. 2016 Sep 22;11(9):e0161249. doi: 10.1371/journal.pone.0161249 (PMC5033568; doi:10.1371/journal.pone.0161249)

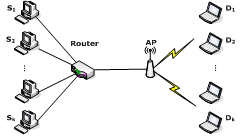

Supplement: S1 File — (ZIP) [file pone.0161249.s001.zip › S1 File/S1 Fig1. The topological graph of Heterogeneous Network.tif]

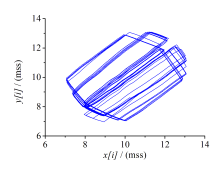

Supplement: S2 File — (ZIP) [file pone.0161249.s002.zip › S2 File/S2 Fig. 2 The attractor in cwnd phase space.tif]

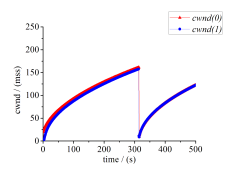

Supplement: S3 File — (ZIP) [file pone.0161249.s003.zip › S3 File/S3 Fig. 3 Two TCP flows with different start time (a)TCP0 begins from 0.1sú1⁄4TCP1 begins from 0.1s.tif]

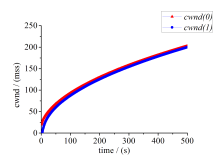

Supplement: S3 File — (ZIP) [file pone.0161249.s003.zip › S3 File/S3 Fig. 3 Two TCP flows with different start time (b)TCP0 begins from 0.1sú1⁄4TCP1 begins from 0.5s.tif]

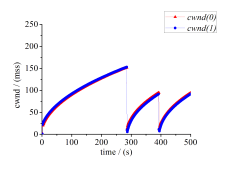

Supplement: S3 File — (ZIP) [file pone.0161249.s003.zip › S3 File/S3 Fig. 3 Two TCP flows with different start time (c)TCP0 begins from 0.1sú1⁄4TCP1 begins from 1s.tif]

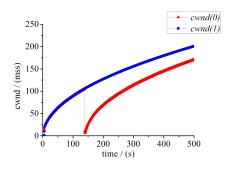

Supplement: S3 File — (ZIP) [file pone.0161249.s003.zip › S3 File/S3 Fig. 3 Two TCP flows with different start time (d)TCP0 begins from 0.1sú1⁄4TCP1 begins from 5s.tif]

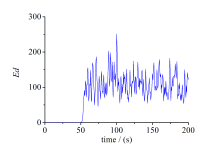

Supplement: S4 File — (ZIP) [file pone.0161249.s004.zip › S4 File/S4 Fig. 4 The difference between the original and modify system.tif]

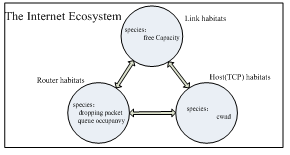

Supplement: S5 File — (ZIP) [file pone.0161249.s005.zip › S5 File/S5 Fig. 5 Internet ecosystem.tif]

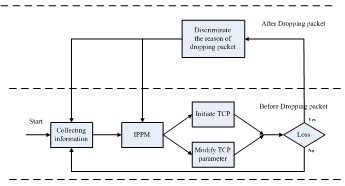

Supplement: S6 File — (ZIP) [file pone.0161249.s006.zip › S6 File/S6 Fig. 6 The structure of improved TCP congestion control mechanism.tif]

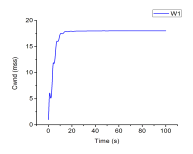

Supplement: S7 File — (ZIP) [file pone.0161249.s007.zip › S7 File/S7 Fig. 7 The evolution for cwnd in proposed algorithm ( a) The change of cwnd for W1.tif]

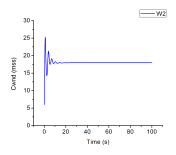

Supplement: S7 File — (ZIP) [file pone.0161249.s007.zip › S7 File/S7 Fig. 7 The evolution for cwnd in proposed algorithm (b) The change of cwnd for W2.tif]

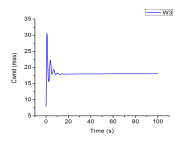

Supplement: S7 File — (ZIP) [file pone.0161249.s007.zip › S7 File/S7 Fig. 7 The evolution for cwnd in proposed algorithm (c) The change of cwnd for W3.tif]

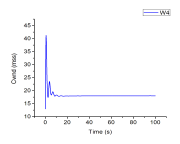

Supplement: S7 File — (ZIP) [file pone.0161249.s007.zip › S7 File/S7 Fig. 7 The evolution for cwnd in proposed algorithm (d) The change of cwnd for W4.tif]

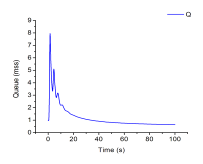

Supplement: S8 File — (ZIP) [file pone.0161249.s008.zip › S8 File/S8 Fig. 8 The evolution of queue for bottleneck link.tif]

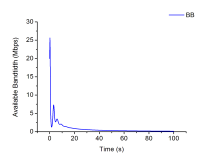

Supplement: S9 File — (ZIP) [file pone.0161249.s009.zip › S9 File/S9 Fig. 9 The evolution of available bandwidth for bottleneck link.tif]

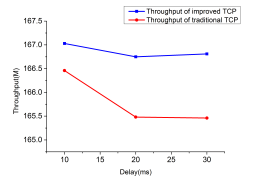

Supplement: S10 File — (ZIP) [file pone.0161249.s010.zip › S10 File/S10 Fig.10 The comparison of throughput for 2 TCP flows when bandwidth=10M.tif]

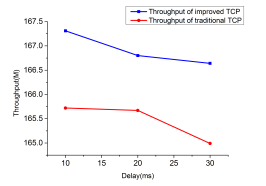

Supplement: S11 File — (ZIP) [file pone.0161249.s011.zip › S11 File/S11 Fig.11 The comparison of throughput for 2 TCP flows when bandwidth=20M.tif]

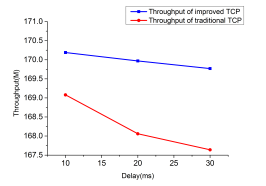

Supplement: S12 File — (ZIP) [file pone.0161249.s012.zip › S12 File/S12 Fig.12 The comparison of throughput for 4 TCP flows when bandwidth=10M.tif]

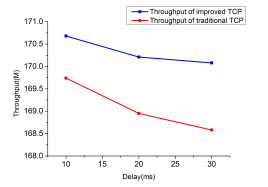

Supplement: S13 File — (ZIP) [file pone.0161249.s013.zip › S13 File/S13 Fig.13 The comparison of throughput for 4 TCP flows when bandwidth=20M.tif]

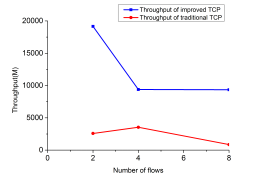

Supplement: S14 File — (ZIP) [file pone.0161249.s014.zip › S14 File/S14 Fig.14 The comparison of throughput for high bandwidth-delay product network.tif]

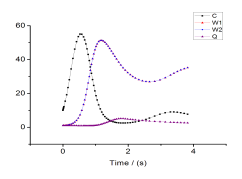

Supplement: S15 File — (ZIP) [file pone.0161249.s015.zip › S15 File/S15 Fig.15 The fairness of two flows.tif]

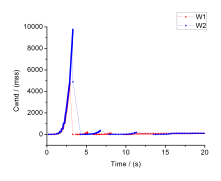

Supplement: S16 File — (ZIP) [file pone.0161249.s016.zip › S16 File/S16 Fig. 16 The changing of cwnd for traditional TCP.tif]

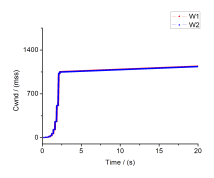

Supplement: S17 File — (ZIP) [file pone.0161249.s017.zip › S17 File/S17 Fig. 17 The changing of cwnd for improved TCP.tif]
